# Understanding the multidimensional cognitive deficits of logopenic variant primary progressive aphasia

**DOI:** 10.1093/brain/awac208

**Published:** 2022-07-20

**Authors:** Siddharth Ramanan, Muireann Irish, Karalyn Patterson, James B Rowe, Maria Luisa Gorno-Tempini, Matthew A Lambon Ralph

**Affiliations:** Medical Research Council Cognition and Brain Sciences Unit, University of Cambridge, Cambridge, UK; The University of Sydney, Brain and Mind Centre and School of Psychology, Sydney, Australia; Medical Research Council Cognition and Brain Sciences Unit, University of Cambridge, Cambridge, UK; Medical Research Council Cognition and Brain Sciences Unit, University of Cambridge, Cambridge, UK; Department of Clinical Neurosciences, Cambridge University Centre for Frontotemporal Dementia, Cambridge, UK; Cambridge University Hospitals NHS Trust, Cambridge, UK; Memory and Aging Center, University of California San Francisco, San Francisco, USA; Medical Research Council Cognition and Brain Sciences Unit, University of Cambridge, Cambridge, UK

**Keywords:** primary progressive aphasia, Alzheimer’s disease, temporoparietal junction, inferior parietal lobe, non-linguistic functions, language

## Abstract

The logopenic variant of primary progressive aphasia is characterized by early deficits in language production and phonological short-term memory, attributed to left-lateralized temporoparietal, inferior parietal and posterior temporal neurodegeneration. Despite patients primarily complaining of language difficulties, emerging evidence points to performance deficits in non-linguistic domains. Temporoparietal cortex, and functional brain networks anchored to this region, are implicated as putative neural substrates of non-linguistic cognitive deficits in logopenic variant primary progressive aphasia, suggesting that degeneration of a shared set of brain regions may result in co-occurring linguistic and non-linguistic dysfunction early in the disease course. Here, we provide a Review aimed at broadening the understanding of logopenic variant primary progressive aphasia beyond the lens of an exclusive language disorder. By considering behavioural and neuroimaging research on non-linguistic dysfunction in logopenic variant primary progressive aphasia, we propose that a significant portion of multidimensional cognitive features can be explained by degeneration of temporal/inferior parietal cortices and connected regions. Drawing on insights from normative cognitive neuroscience, we propose that these regions underpin a combination of domain-general and domain-selective cognitive processes, whose disruption results in multifaceted cognitive deficits including aphasia. This account explains the common emergence of linguistic and non-linguistic cognitive difficulties in logopenic variant primary progressive aphasia, and predicts phenotypic diversification associated with progression of pathology in posterior neocortex.

## Teaser

In a new clinico-anatomical model, Ramanan *et al.* integrate advances from cognitive neuroscience and neuropsychology to propose a role for left temporoparietal degeneration in underpinning emergent linguistic and non-linguistic cognitive deficits in logopenic variant primary progressive aphasia.

## Introduction

Primary progressive aphasia (PPA) refers to a group of neurodegenerative disorders affecting language functions in early stages, due to degeneration of a distributed, largely left-lateralized language network.^1,2^ Historical descriptions of PPA-like syndromes can be found in late 19th and early 20th centuries in reports by Pick, Sérieux, Dejerine, Rosenfeld and others.^1^ Contemporary clinical, anatomical and pathological understanding of PPAs has evolved greatly in the last 40 years. Key to this resurgence was a seminal case series by Mesulam^3^ describing progressive aphasia with left perisylvian atrophy and slow rates of disease progression. Marked general cognitive decline emerged only later in the disease course.^4^ Subsequent classifications of PPA have expanded the phenotype to include patients with motor-speech planning, grammatical and semantic memory deficits, identifying three main clinical variants: a ‘non-fluent/agrammatic’ variant (nfvPPA) characterized by motor-speech dysfunction and/or agrammatism stemming from primary fronto-insular degeneration;^5^ a ‘semantic’ variant (svPPA, also called semantic dementia) presenting with degradation of conceptual knowledge due to anterior temporal degeneration;^6^ and a third ‘logopenic’ variant (lvPPA)^7^ that forms the focus of the current Review. Clinically, lvPPA patients display slowed, disrupted spontaneous language production marked by word-finding pauses, anomia, phonological errors and poor length-dependent repetition of sentences, amid relatively preserved grammatical processing, motor speech (unlike nfvPPA) and semantic knowledge (unlike svPPA).^2,8^ Comprehension of long sentences is also affected and, in part, could arise from poor phonological short-term memory.^8–10^ This constellation of language difficulties emerges from primary degeneration of the left-lateralized posterior temporal gyri, temporoparietal junction and inferior parietal lobes (TPJ/IPL) and their interstices.^8,11^ Recent advances in neuroimaging of disease progression in PPA syndromes conceptualize them as network-level disorders, in which neurodegeneration starts in a syndrome-specific susceptible epicentre and then spreads in a predictable, constrained manner along structurally/functionally connected brain regions.^12,13^ Accordingly, with disease progression, atrophy in lvPPA encroaches into regions in the left hemisphere that are functionally coupled with TPJ/IPL (via language and Default Mode networks), followed by regions in the right hemisphere.^14–19^

To date, the lens of lvPPA clinical investigations has largely focused on language profiles, mainly because, by definition, the main presenting clinical complaint is that of language disturbances. However, a growing number of studies pose an important diagnostic challenge: that numerous lvPPA patients display concurrent, non-linguistic cognitive deficits on neuropsychological testing. Of note, even in Mesulam’s early PPA cases who probably had the logopenic variant, calculation difficulties were noted during clinical presentation.^3^ When non-linguistic cognitive functions are tested in depth, most lvPPA cases appear to exhibit moderate-to-marked levels of non-linguistic cognitive dysfunction.^20^ Others find that the severity of non-linguistic difficulties in lvPPA may parallel the severity of aphasia and emerge relatively independent of disease severity.^21^ This issue is complicated by overlaps in both language and non-linguistic cognitive performance between lvPPA, amnestic Alzheimer’s disease and posterior cortical atrophy: three clinical entities often sharing common pathology.^16^ Piecing together the diagnostic and mechanistic puzzle of why lvPPA presents with linguistic and non-linguistic cognitive difficulties is vital to ensure accurate diagnosis, characterization and prognostication.

In this paper, we propose a new clinico-anatomical framework that encompasses the ‘multidimensional’ deficits in lvPPA. The combination of core language and variable non-linguistic deficits may arise from shared neurophysiological mechanisms, and the nature and localization of neurocognitive processes supported by the left TPJ/IPL region—the epicentre of atrophy in the syndrome. The contribution of this region and functionally connected areas to language disruption in lvPPA has received significant attention, but its role in cognitive processes beyond language in this syndrome has been relatively overlooked. By integrating insights from contemporary cognitive neuroscience and neuropsychology, we review the evidence of domain-general and domain-selective mechanistic roles for these regions. By then reversing these inferences, we can consider the phenotypic outcome of left TPJ/IPL degeneration to lvPPA, updating our understanding of why some patients present with a relatively pure aphasic profile while others display additional non-linguistic dysfunction.

To be clear from the outset, our goal of this Review is not to rewrite the current diagnostic criteria for lvPPA, but rather to explain phenotypic diversification in the syndrome with implications to its accurate diagnosis and timely introduction of multidomain symptomatic treatments.

## Insights from contemporary cognitive neuroscience

We propose that patterns of verbal and nonverbal cognitive difficulties observed early in the lvPPA disease course are a direct reflection of (i) the cognitive complexity of TPJ/IPL; and (ii) the amount and distribution of neuropathology, atrophy and/or hypometabolism in TPJ/IPL and structurally/functionally connected regions and networks.^10,22–25^ The impact of TPJ/IPL pathology in lvPPA may further cause disruptions in cognitive processing related to up/downstream regions, such as the hippocampus and right IPL, and connected functional networks, compounding cognitive dysfunction in the syndrome. The current proposal does not exclude contributions of other brain regions to cognitive dysfunction in lvPPA; but in this Review, we focus on TPJ/IPL due to its complex cognitive roles and early involvement in this syndrome. A detailed review of left TPJ/IPL functionality is beyond the scope of our Review^26–29^; however, by considering key themes regarding its neurocognitive organization, we can provide testable neural hypotheses explaining the multifaceted cognitive presentation of lvPPA.

Historical cognitive neuroscience and neuropsychology evidence has implicated left TPJ/IPL in multiple, apparently distinct, cognitive tasks. For instance, Gerstmann’s syndrome, emerging from left IPL damage, encompasses both linguistic (dysgraphia and occasionally, aphasia) and non-linguistic (finger agnosia, dyscalculia, left-right disorientation) complaints.^30^ Functional neuroimaging suggests that the multidomain involvement of TPJ/IPL can be traced to its role in deploying key neurocomputational resources to meet demands of different cognitive tasks.^28,31^ Three principal features of this region underpin its domain-general and domain-selective functions:

- Neuroanatomically, TPJ/IPL are segregated from primary sensory cortices. This absolves them from processing dynamic fluctuations in the immediate sensory environment, and facilitates the processing of multimodal information and diverse cognitive domains.^28,32,33,29,34^
- Computational models suggest that repeat processing, integration and buffering of information can promote extraction of time-, space- and context-invariant representations that can be repurposed to different task demands.^28,35,36^ TPJ/IPL harbour this functional capacity, whereas TPJ/IPL lesions characteristic of semantic aphasia impair the use of time- and context-appropriate information across semantic and non-semantic domains.^37,38^
- Functional specialization within TPJ/IPL is not sharply fractionated, but varies between subregions in a graded manner and is tied closely to structural/functional connectivity patterns of each subregion. This characteristic is seen in the temporal granularity of information being processed here. For example, cognitive tasks processed over shorter time periods (e.g. phonology, sound) preferentially recruit anterior TPJ/IPL regions (e.g. supramarginal gyrus) whereas posterior IPL (e.g. angular gyrus) is preferentially recruited in tasks reliant on longer temporal windows, where processing of accumulating information is required (e.g. sentence and narrative processing, episodic memory retrieval).^28,34,39^

**Figure 1:**
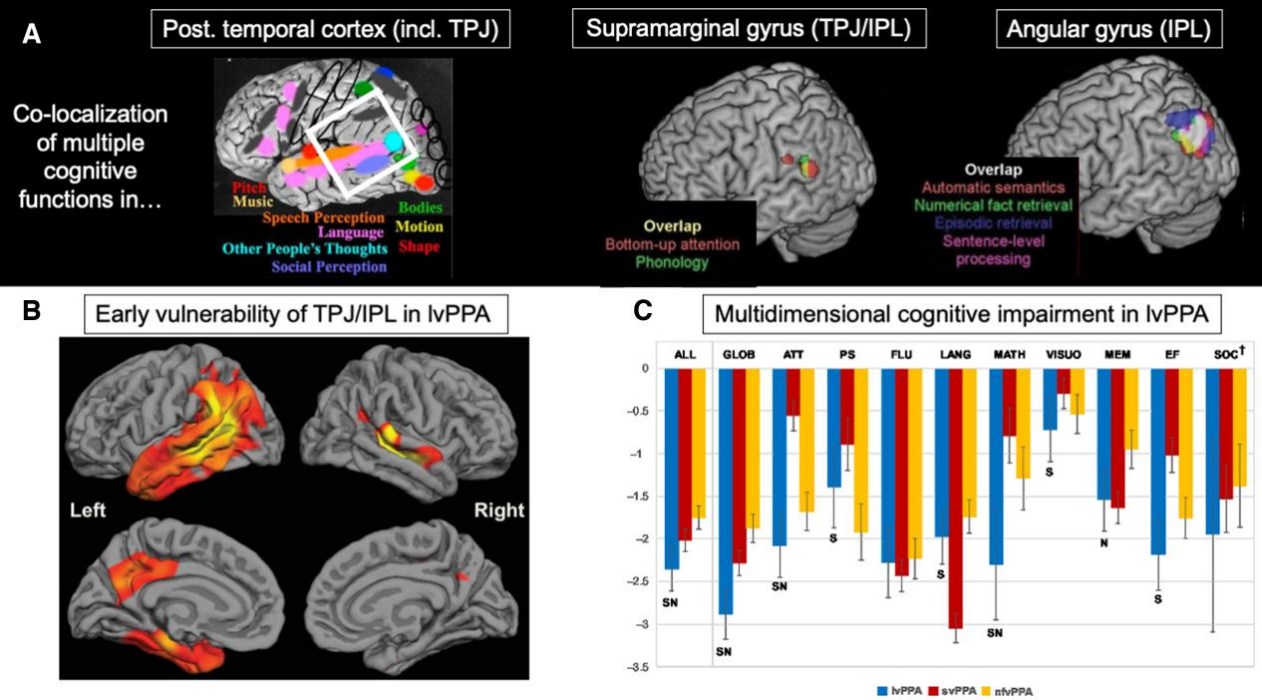
**Graphical summary of the main premise of this Review.** (**A**) Insights from cognitive neuroscience indicate that the left posterior temporal cortices (including superior/middle/inferior temporal gyrus and TPJ; together, denoted within the white square) (reprinted from Kanwisher^40^) and left supramarginal and angular gyri (components of the IPL) (reprinted from Humphreys and Lambon Ralph^28^) support multiple cognitive functions, possibly through deployment of domain-general and domain-selective computations. These computations support phonology and working memory, episodic and semantic memory, social and numerical cognition, visuospatial and executive abilities, as well as attention and praxis functions. (**B**) lvPPA targets left posterior temporal and TPJ/IPL regions (depicted here as reduced cortical thickness, i.e. warmer colours) suggesting the aforementioned cognitive functions dependent on TPJ/IPL functionality should be affected (reprinted from Leyton *et al.*^11^ with permission from IOS Press). (**C**) A recent meta-analysis of neuropsychological performance in 663 lvPPA patients (across 51 studies) found significant performance deficits across standardized neuropsychological measures of episodic memory, social and numerical cognition, executive functions, and attention in lvPPA relative to nfvPPA (‘N’ in figure) and svPPA (‘S’ in figure) groups (reprinted from Kamath *et al.*^21^ with permission from Cambridge University Press), showcasing the multidimensional cognitive profile of this syndrome.

On the basis of these principles, we evaluate evidence for TPJ/IPL in multidomain cognition, with relevance to lvPPA (Fig. 1A–C).

Considering language functions, posterior superior temporal and inferior supramarginal gyri are involved in extraction of spectro-temporal features of sound^41^ and extraction and maintenance of phonological representations.^42^ These specific functions, in turn, may be supported by shared neurophysiological mechanisms within TPJ/IPL. For example, the planum temporale acts as an auditory ‘computational hub’, disambiguating incoming auditory information, matching these representations to existing auditory templates (derived from experience), in turn, supporting its transformation into subsequent motor programmes for different cognitive/behavioural domains (e.g. object recognition, emotional vocalization).^43–45^ Ventral and posterior portions, including posterior middle temporal regions, are associated with multimodal semantic-executive related processing,^46–48^ while angular gyrus participates in constructive elements of verbal episodic recollection,^49–51^ sentential and combinatorial processing^52^ and buffering sequential information in coherent contexts.^53,54^ These functional specializations are corroborated by extant neuropsychological evidence. For example, conduction aphasia, emerging from damage to the inferior supramarginal gyrus and underlying arcuate fasciculus, specifically disrupts speech repetition but spares semantic-executive control.^55,56^ Conversely, transcortical sensory aphasia and semantic aphasia, occurring from lesions in the middle/posterior cerebral artery watershed territory affecting posterior middle temporal regions (outside the supramarginal gyrus), display poor semantic-executive processing but relatively intact phonological functions.^38,57^ Finally, combined damage to the entire left TPJ/IPL, such as in Wernicke’s aphasia, disrupts both phonological and semantic-executive control functions, irrespective of tested modality.^58^

For non-linguistic domains, left IPL emerges at the nexus of multiple non-linguistic cognitive capacities. As per our third principle (i.e. functional gradation within TPJ/IPL for processing information varying in temporal granularity), the anterior IPL (supramarginal gyrus) shows involvement in tasks requiring relatively shorter temporal processing windows, such as directing bottom-up attention to external stimuli and internally generated thought (e.g. episodic recollection).^59^ Longer temporal windows in posterior IPL (angular gyrus) facilitate its involvement in transformation and integration of accumulating information, irrespective of its nature or modality.^28,54^ For example, during memory recollection and future-oriented thinking: (i) angular gyrus and medial temporal regions show functional coupling; and (ii) angular gyrus activity scales with the vividness, strength and accuracy of retrieved memories, and shows demonstrable sensitivity to integration of multimodal information.^49,50,60,61^ Accordingly, primary IPL damage, irrespective of aetiology, impairs performance on cognitive endeavours requiring mental simulations, including episodic retrieval and scene construction,^62–64^ theory of mind and social cognition^65^ and spatial navigation.^66^ Angular gyrus is further integral to transformation and retrieval of numerical knowledge,^67^ explaining dyscalculia following IPL damage in Gerstmann’s syndrome. The integration role of the IPL further extends to motor behaviour, where supramarginal gyrus directs attention to motor sequences^68^ while supramarginal and angular gyri together aid integration of individual motor goals and their transformation into external motor actions.^69^ This particular finding implicates the IPL as a neural substrate of ideational apraxia as well as volitional apathy.^69,70^ Taken together, the evidence points to a role for the TPJ/IPL in supporting multiple cognitive processes spanning language and beyond.

## lvPPA clinical observations mirror cognitive neuroscience

Reverse inference from the cognitive neuroscience and neuropsychology literature on the nature of multidomain cognitive processes of the TPJ/IPL region (reviewed previously, see also Fig. 1) provides a potential foundation for explaining the core language and co-occurring variable cognitive deficits in lvPPA in terms of stochastic spreading of pathology and atrophy from the TPJ/IPL epicentre.

### A prototypical language deficit

The core language deficit of lvPPA is in keeping with the role of the posterior superior temporal gyrus/inferior supramarginal gyrus in phonological processing and working memory. In lvPPA, errors of phonology and lexical retrieval during word formation emerge from dysfunction of these specific regions.^11,71,72^ Difficulties in decoding phonemic structure in lvPPA patients further relate to attenuated activation of superior temporal regions as noted on functional neuroimaging.^73^ With disrupted phonological short-term memory, length-dependent repetition deficits are a canonical feature of lvPPA, manifesting in a profound inability to retain, manipulate and reproduce long segments of verbal information (e.g. repeating lengthy words, sentences and letter/digit strings in forward/reverse orders).^74,75^ On this view, the language and anatomical profile of lvPPA most closely resembles conduction aphasia post-stroke,^74,76,77^ although it must be acknowledged that the major PPA syndromes have either little or only partial analogues in vascular aphasiology, in terms of overall linguistic and cognitive performance.^78^ If and when the underlying pathology encroaches into anterior and ventral temporal cortices, semantic comprehension impairments, such as those in svPPA, are expected.^79,80^ Similarly, if the disease spreads into fronto-insular regions and connecting arcuate/superior longitudinal fasciculus, then motor-speech difficulties (resembling those observed in nfvPPA) may become part of the clinical profile.^81^

### The multidimensional non-linguistic deficits

Relative to healthy controls and other PPA variants, neuropsychological investigations of well-characterized lvPPA patients identify deficits in processing speed, executive function, sustained attention, working memory and visuospatial function.^82–85^ Dyscalculia as early as 1 year post disease onset, and emergence of apraxia within 2 years are also reported.^7,86^ A recent case report of an lvPPA patient with canonical TPJ/IPL atrophy noted attentional and visuospatial complaints (manifesting as hemispatial neglect)^87^—a constellation of complaints typically seen in posterior cortical atrophy following superior/inferior parieto-occipital neurodegeneration. Another case report of a patient meeting lvPPA clinico-radiological criteria evidenced deregulated semantic-executive control when using electrical appliances, wherein the patient ‘clearly understood what these appliances are and what they are used for, but was not sure how to use them’.^86(p2)^. This profile appears to fulfil standard definitions of ideational apraxia, and is highly reminiscent of semantic control deficits in semantic aphasia (who present with concurrent ideational/dysexecutive apraxia),^88–90^ where damage to inferior/middle posterior temporal and IPL (angular gyrus) regions results in context-invariant executive deregulation of verbal and nonverbal semantic information.^38,89,90^ In this regard, probing the status of semantic control functions in lvPPA forms an important line of inquiry as it may inform whether deregulated semantic control partly explains the co-occurrence of some non-linguistic symptoms, such as apraxia, in this syndrome.

Given the role of the angular gyrus in episodic memory processing, atrophy/pathological deposition to this region can impair episodic retrieval.^34,49,60,91^ Accordingly, an additional under-appreciated cognitive complaint in lvPPA is episodic amnesia, closely linked to IPL involvement in the syndrome. By the time of their first clinic appointment, a third of patients report misplacing objects, missing appointments, getting lost and facing difficulty in learning new tasks.^92,93^ Both carer- and clinician-indexed reports further attest to daily memory difficulties.^94^ On objective standardized neuropsychological memory assessment, verbal and nonverbal episodic memory deficits in lvPPA can be prominent to a magnitude comparable to amnesic Alzheimer’s disease and are associated with changes to integrity of the bilateral IPL (angular gyri) and their structural connections to memory processing regions in the medial temporal lobe (e.g. hippocampus).^94–96^ Episodic amnesia in lvPPA can also extend to remote autobiographical memories encoded decades before the onset of clinical symptoms (e.g. from teenage and early adulthood years), with poor autobiographical recall, irrespective of the temporal epoch of the memory, correlating with angular gyrus involvement in the syndrome.^97^

Beyond episodic memory, lvPPA patients show difficulties on tasks of nonverbal auditory object processing (e.g. phoneme discrimination, prosody perception, global pitch and timbre processing), in the absence of peripheral hearing difficulties.^98–101^ These impairments have been found to be closely associated with dysfunctional working memory,^98,100^ further correlating with integrity of the left IPL and parietal cortices.^100,101^ Spatial working memory deficits are also prominent in lvPPA, again associated with degeneration of superior parietal cortices and their functional connections with prefrontal brain regions.^102–104^ Across clinical variants of Alzheimer’s disease (including lvPPA), increased pathological accumulation in TPJ/IPL and parietal cortex strongly correlates with emergent impairments of episodic and semantic memory, plus executive and visuospatial functions.^105^ In lvPPA particularly, patients displaying greater visuospatial, executive and general cognitive disturbances typically show intensified atrophy to left IPL/superior parietal regions.^20,106^ Corroborative evidence also comes from studies adopting the inverse ‘imaging-first’ classification approach, where lvPPA patients with greater left IPL/parietal atrophy/hypometabolism display poorer nonverbal memory, visuospatial and executive performance, in contrast to lvPPA patients with temporal-dominant atrophy/hypometabolism.^107^ Finally, there is some evidence for behavioural dysfunction in lvPPA, such as increased apathy, anxiety, irritability, agitation and difficulties in emotion detection.^108–112^ Ultimately, it is unsurprising that a combination of aphasia, poor memory and general cognition, increased apathy and agitation significantly compounds carer burden and caring-related challenges in lvPPA.^110,113,114^ Additional investigations of the inter-dependence between behavioural and mood symptoms, the neural bases of non-linguistic cognitive and behavioural difficulties, and how these changes relate to underlying pathological and genetic changes in lvPPA are required (see Box 1). Nevertheless, the extant evidence consistently implicates TPJ/IPL dysfunction as one of the brain regions central to multidimensional cognitive decline in the syndrome. This anatomical framework also potentially explains the development of incipient lvPPA-like features in other Alzheimer’s disease clinical variants, especially in early onset atypical presentations such as posterior cortical atrophy,^92,131,132^ as the underlying pathology encroaches into TPJ/IPL.

**Box 1 Links between multidimensional cognitive impairment, underlying Alzheimer’s disease pathology and progranulin gene mutations in lvPPA.** Clinicopathological associations are key to achieving accurate prognosis and treatment decisions. Over 80% of lvPPA patients have underlying Alzheimer pathology, mostly noted as amyloid-positive PET scans.^16,115–118^ More recently, reports have emerged of cases showing an lvPPA-like clinical picture amid amyloid-negative profiles. In these cases, a common finding is the mutation of the progranulin gene, which is typically associated with frontotemporal lobar degeneration syndromes.^119–122^ In addition to the classic linguistic profile of lvPPA, many such cases display concurrent difficulties in reading, episodic memory, executive functions and calculation,^123,124^ suggesting links between cognitive multidimensionality and underlying neurobiological and genetic factors. In current neurodegenerative dementia clinico-pathological conceptualizations, lvPPA is considered as an atypical clinical variant of Alzheimer’s disease.^16^ Therefore, an lvPPA patient with an amyloid-positive scan, a prototypical aphasic profile, poor memory, visuospatial and/or executive dysfunction may rule in several different diagnostic labels including mixed PPA, advanced amnesic Alzheimer’s disease and an Alzheimer’s disease clinical variant with visuospatial and/or language features. As the syndrome straddles clinicopathological boundaries of both PPA and Alzheimer’s disease taxonomies, it is sensible to ask whether multidimensional cognitive difficulties in lvPPA are parsimoniously explained by underlying pathological and/or genetic mechanisms.

Given the paucity of evidence on correspondence between clinicopathological and cognitive performance data in lvPPA, a direct answer to this question is currently hard to establish. For example, one study noted that PPA with Alzheimer’s disease pathology (most of whom met a clinical diagnosis of lvPPA) is associated with episodic memory preservation, despite these patients presenting significant TPJ/IPL and medial temporal atrophy.^125^ On the other hand, accumulating evidence suggests that lvPPA patients with and without amyloid-positive profiles perform comparably on standardized measures of lexical retrieval, speech and language, executive function, processing speed and verbal memory.^126–129^ Comparable profiles between high- and low-amyloid burden lvPPA patients are also evident on visuospatial and non-verbal memory tasks, even when disease severity and overall language status are accounted for.^127,130^ Moreover, data-driven clustering solutions of lvPPA cognitive performance find no significant differences in amyloid burden, demographic and disease duration indices between lvPPA with mild, marked or no co-occurring general cognitive deficits.^20^ Likewise, when contrasting lvPPA cases with and without progranulin gene mutations, comparable language, cognitive, and neuroanatomical profiles (on structural MRI) are notable.^119,120^ Due to the rarity of such reports, more work is required on this front. However, most findings suggest that the pathological or genetic profile, by itself, may not be sufficient to explain variations in non-linguistic cognitive difficulties in lvPPA. Rather, as we propose, the key factor in variable cognitive profiles early in the syndrome may be the extent to which the distribution and loading of pathology and atrophy/hypometabolism are localized in the TPJ/IPL and neighbouring regions.

## Other contributory mechanisms

### Non-linguistic deficits and their relationship with primary aphasia

Primary verbal degradation in lvPPA greatly influences performance on tasks taxing working memory and phonological abilities, such as reading, verbal fluency and verbal episodic memory.^133–135^ These deficits often interfere with patients’ ability to understand complex verbal instructions. However, there is compelling evidence that many nonverbal cognitive deficits observed in lvPPA are not simply a reflection/by-product of the patients’ progressive aphasia. Patients with lvPPA can show marked deficits on cognitive functions that circumvent language demands, such as nonverbal episodic memory, nonverbal auditory object processing, spatial orientation, spatial working memory and visuospatial functioning.^83,96,98,99,136,137^ Likewise, lvPPA patients exhibit impaired performance on verbal memory, and visuospatial and executive processing tasks, even after statistically controlling effects of aphasia severity.^94,97,106,109^ In this regard, detailed lvPPA case reports are particularly insightful. In one report, the earliest recorded complaints involved calculation and sustained attention difficulties occurring a few months prior to the emergence of a florid lvPPA aphasic profile.^87^ In another study, Pozzebon *et al*.^138^ interviewed spouses of five lvPPA patients and found all carers to report incipient apathy and social withdrawal, plus deficits of episodic memory and sustained attention emerging almost 1–3 years before spousal recognition of frank expressive language deficits. These deficits were not core of the patients’ presentation, which was of language impairment, thus meeting the primary criteria for progressive aphasia. These findings indicate that non-linguistic deficits in lvPPA can emerge independent of and in parallel to primary aphasia, even where the patient’s core complaint is that of language difficulty.

### Non-linguistic deficits emerge with disease progression

As with other progressive disorders, relatively ‘pure’ cases with circumscribed clinico-anatomical presentation evolve to show clear and increasing multidomain dysfunction as the underlying pathology propagates. Although longitudinal profiles of some lvPPA patients neatly fit this pattern,^139^ this hypothesis is not a sufficient explanation the early non-linguistic deficits in many other people with lvPPA. Even at the mildest clinician-indexed disease stages (mean Clinical Dementia Rating score of 0.5–0.7), difficulties in calculation and spatial working memory can be evident in lvPPA.^7,8^ Likewise, poor nonverbal episodic memory performance remains prominent in lvPPA, even after statistically controlling for disease severity.^94^ The confounding issue of disease severity has been addressed by recent data-driven studies parcellating its effects. For example, when overall language performance is employed as a categorical proxy for disease severity, the magnitude of general cognitive impairment scales with aphasia severity.^79,140^ A key limitation of this approach is that categorizing lvPPA patients first on aphasia severity and then on non-linguistic cognitive performance overlooks linguistic and non-linguistic difficulties occurring in parallel. Using step-wise classifications, therefore, may bias our determination of the absence/magnitude of non-linguistic complaints.

**Figure 2:**
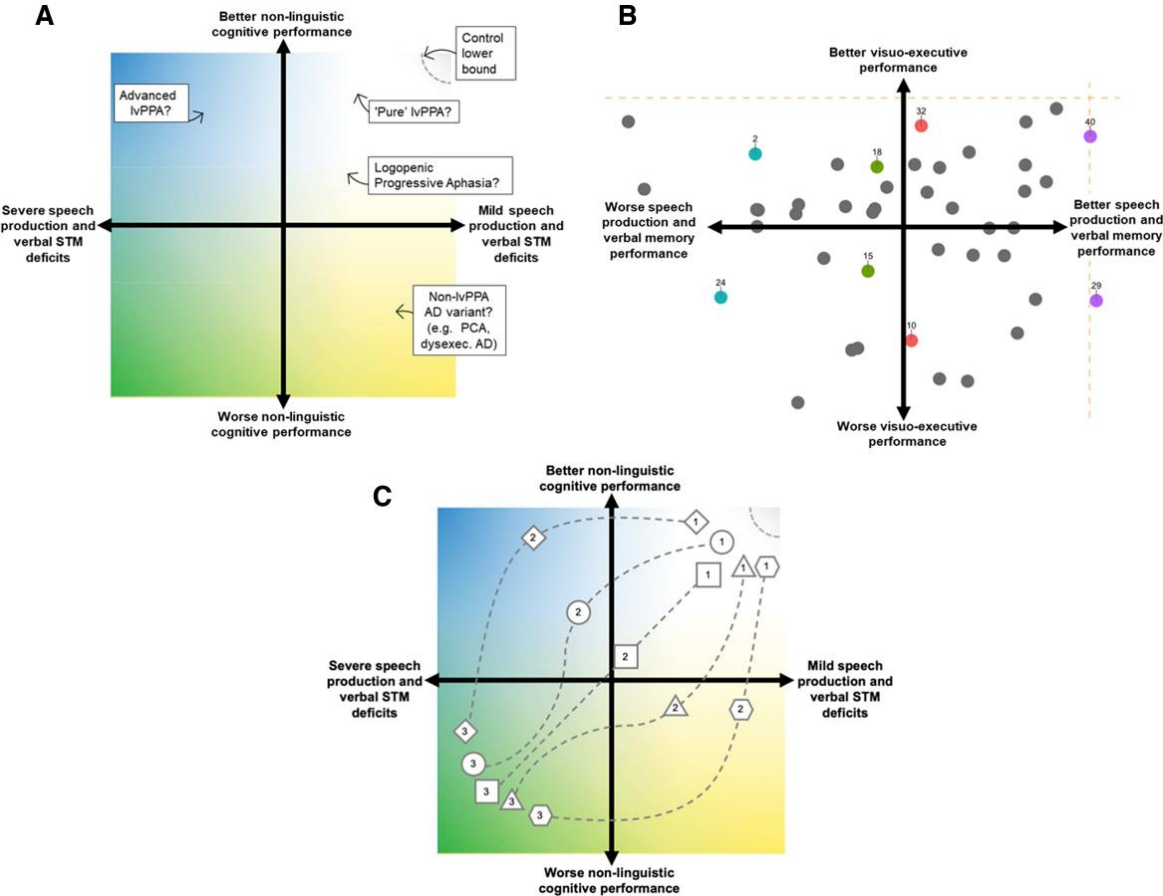
**Capturing phenotypic variations in lvPPA within a graded, multidimensional space.** (**A**) Capturing cognitive changes to language and non-linguistic domains within a multidimensional space allows an examination of the graded, individual-level variations in patients’ profiles, as well as the underlying neural machinery that is affected in each case. STM = short-term memory; PCA = posterior cortical atrophy; AD = Alzheimer’s disease. (**B**) The nature of such a multidimensional graded space including the lvPPA phenotype receives support from the empirical findings of Ramanan *et al*.^106^ Here, principal component analysis on comprehensive neuropsychological performance data (*n* = 43 lvPPA) revealed multiple, independent sources of variation, captured best by two orthogonal dimensions of performance labelled ‘speech production and verbal memory’ and ‘visuospatial and executive’ (visuo-executive) performances. The finding that lvPPA patients, irrespective of the extent of their speech production and verbal memory performance, display some level of visuo-executive changes (c.f. coloured pairs) suggests individual-level variation in cognitive phenotype that necessitates the use of a multidimensional space. Here, dashed gold lines indicate lower bound of performance in healthy Controls (adapted from Ramanan *et al.*^106^). (**C**) The adoption of a multidimensional space to account for linguistic and non-linguistic changes in lvPPA can further afford mapping of heterogeneous disease trajectories dynamically varying at the individual-level. This includes individuals showing generalized cognitive impairment that are (i) linear with disease progression (square); (ii) emerge quickly into disease onset (triangle and pentagon); or (iii) emerge slowly with disease advancement (diamond and circle). Here, numbers correspond to the clinical assessment round (1 = 1st assessment etc.). STM = short term memory.

A recent study overcame this limitation by using principal component analysis in 43 well-characterized lvPPA patients, at varying disease stages, who had undergone comprehensive multidomain neuropsychological testing.^106^ The principal component analysis revealed multiple, concurrent sources of variance across neuropsychological performance in lvPPA. Extraction of orthogonal dimensions of performance variation allowed (i) exploration of confounds associated with disease and aphasia severity that otherwise pervade across measures; and (ii) situation of patients along independent dimensions of verbal and nonverbal cognitive performance thereby capturing their graded, inter-individual variations in cognitive impairment. The results indicated that the cognitive profile of lvPPA is best characterized along two orthogonal dimensions: (i) speech production and verbal memory deficits; and (ii) visuospatial and executive changes (Fig. 2B). Supporting our proposed framework, reduced integrity of left IPL and right fronto-parietal grey matter was characteristic of lvPPA cases with poorer visuo-executive performance. Three results from this study argue against the hypothesis that disease severity is the primary modulator of general cognitive deficits in lvPPA: (i) language and non-linguistic performance did not align with a single ‘disease severity’ factor but lay on orthogonal dimensions; (ii) the visuo-executive factor correlated weakly with independent clinical measures of disease severity and disease duration; and (iii) visuo-executive deficits were present systematically across the lvPPA cohort, irrespective of the magnitude of speech production difficulties.^106^ These findings are consistent with the case studies reporting concurrent verbal and non-verbal dysfunction early in the disease course; and formally show that multidimensional nonverbal cognitive difficulties are systematically present and form an early and integral part of the symptom complex of lvPPA.

## Accommodating lvPPA cognitive variations within a graded multidimensional phenotypic space

Given the evidence for multidomain cognitive dysfunction, it has been argued whether the term ‘lvPPA’ be reserved for ‘pure’ cases (impaired single-word retrieval and sentence/phrase repetition without concurrent non-linguistic impairment), while ‘Logopenic Progressive Aphasia’ might better capture individuals displaying a dynamic, multifaceted phenotype beyond the linguistic constraints of a pure PPA.^125^ Terminological distinctions are non-trivial; terminologies themselves are carefully derived and require universal consensus. Although language difficulties remain the primary presenting complaint for these patients, the evidence reviewed here might bring into question (i) whether additional non-linguistic cognitive dysfunction is representative of the syndromic designation of lvPPA; (ii) whether ‘logopenic progressive aphasia’ might better reflect the cognitive profile of such patients; or (iii) whether such cases may represent a new subtype/subcategory of lvPPA. How do we then best capture these terminological differences and inter-individual differences in cognitive performance?

As per recent developments in post-stroke aphasia and frontotemporal dementia studies,^78,141–143^ we propose that cognitive changes in individual lvPPA patients are best conceptualized as graded, individual-level differences within a multidimensional space (Fig. 2A). By this view, differences between two lvPPA patients in non-linguistic cognition do not necessarily reflect a sharp categorical distinction in their clinical conceptualization. Instead, these differences reflect graded, individual-level variations directly tied to disease encroachment into different brain regions. Importantly, embracing a graded, multidimensional approach does not require discarding current categorical labels. Instead, categorical labels serve as direct pointers to a specific region within this multidimensional space (Fig. 2A).

To simplify, we can use a colour analogy to represent this multidimensional space, with each axis representing systematic variation on verbal and nonverbal cognitive performance (Fig. 2A) as has been indicated by previous evidence (Fig. 2B).^106^ In this space, some cases may be ‘yellow’, others ‘blue’ and many others various intersections of these colours. In this hue space, ‘pure’ lvPPA patients (with prototypical aphasia and atrophy centred in posterior superior temporal gyrus/supramarginal gyrus) may represent a subsample of individuals within this larger space. What is important to recognize is that ‘pure’ lvPPA as a clinical entity does not occupy the entire multidimensional space, but rather locates in an area of a larger space spanning variable clinical presentations of the syndrome. Variations in atrophy/functional disruptions within the TPJ/IPL cortices, and connected regions, will generate graded variations in the type and severity of the individual verbal and nonverbal profile, determining locations of individuals within this space. In short, phenotypic deviations from prototypical lvPPA may be graded rather than absolute, and reflect the variations in local pathology (Fig. 2A).

The adoption of such a multidimensional space may further afford the mapping of dynamic cognitive devolution of individual lvPPA patients, tied closely to patterns of disease encroachment (Fig. 2C). It is proposed that, if phenotype can be multidimensional, disease progression can be too and the adoption of such a space can accommodate fluid and heterogeneous cognitive degradation patterns. This includes individuals showing generalized cognitive impairment that emerge (i) linearly with disease and aphasia progression (Fig. 2C, square); (ii) quickly into disease onset (Fig. 2C, triangle and pentagon); or (iii) slowly with disease advancement (Fig. 2C, diamond and circle). This space further enables comparison of different clinical syndromes (lvPPA, amnestic Alzheimer’s disease, posterior cortical atrophy) to derive transdiagnostic understandings of symptom progression. Ultimately, we propose that clinical subtypes of Alzheimer’s disease can be coherently conceptualized as displaying graded patterns of cognitive variation, mainly dependent on the neuroanatomical regions of impact.^23,106,144^ This proposal holds important clinical implications, especially when patients present with co-occurring language, memory and visuospatial difficulties that each require recognition and management. It holds importance for how individuals are sampled for research and clinical studies as ‘representative’ of a clinical condition, as the subspace from where we sample directly determines emergent results. This approach can also accommodate discrepancies regarding usage of the terminologies in published literature, such as ‘lvPPA’ versus ‘Logopenic Progressive Aphasia’ to refer to patients with prototypical lvPPA clinico-anatomical profiles, but with varying, graded levels of additional non-linguistic difficulties.

## Conclusions

The evidence for non-linguistic cognitive deficits in lvPPA does not necessitate a change in current diagnostic criteria. Rather, the combination of linguistic and non-linguistic deficits indicates a need to re-evaluate the phenotype and pathophysiology, and consider the use of non-linguistic deficits in the diagnostic and management plan of lvPPA. We foresee three direct clinical advantages to this change.

First is the need to assess both language and non-linguistic features in PPA diagnosis. Naming, spontaneous speech and paraphasic/lexical error patterns, as individual measures, are increasingly recognized as providing poor specification of PPA type.^78,126,145,146^ On language performance alone, specific expertise is necessary to distinguish lvPPA from other PPA types^78,147^ and the clinical accuracy of language measures in differentiating PPA variants may not always improve upon the incorporation of additional pathological information.^126,127^ This complicates identification of the syndrome and, to the untrained clinical eye, risks conferring an lvPPA diagnosis through eliminating nfvPPA and svPPA labels. In contrast, recent work suggests that non-linguistic measures, such as nonverbal memory, may hold >80% accuracy in distinguishing lvPPA from other non-fluent variants (if not from amnestic Alzheimer’s disease).^94^ While the discriminative use of other non-linguistic tests remains to be explored, co-reliance on linguistic and non-linguistic measures uniquely stressing functions supported by TPJ/IPL may significantly improve accurate diagnosis of lvPPA. For practising clinicians, detailed testing of verbal and nonverbal cognition in the syndrome can improve understanding of the structure of this multidimensional space and help identify measures that are highly representative of each axis of space. In turn, this information can be used to cull lengthy test batteries and retain those efficient and effective measures that capture the most important cognitive-behavioural variation in lvPPA. The success of this method has recently been demonstrated in post-stroke aphasia^148^; how informative it is in PPA, where aetiology, cognitive performance and progression patterns are complex and highly variable, remains to be better understood. At the point of diagnosis, specific quadrants/locations within this space may also inform selection, titration and timing of management approaches, therapies and treatments, as well as inclusion and stratification of specific individuals into trials.

Second, given their importance in the multifaceted clinical profile of lvPPA, TPJ/IPL and parietal cortex seem likely to become neuroanatomical targets of symptomatic intervention in this syndrome. In this regard, recent work has revealed links between excitatory neurostimulation of prefrontal regions and facilitation of oral and written language improvement in lvPPA.^149,150^ As for the IPL, emerging evidence in PPA indicates that a combination of IPL neurostimulation and speech and language training can induce (i) improved naming and verbal fluency performance; (ii) transfer of gains to unlearnt items and select cognitive domains (e.g. overall digit span performance); and (iii) benefits sustaining for up to 2 weeks.^151,152^ These findings indicate the need for more studies exploring interventions targeting the parietal cortex for linguistic, non-linguistic cognitive and functional improvement in lvPPA. Moreover, TPJ/IPL and general parietal dysfunction are largely considered to form the common neuroanatomical denominator associated with phenotypic diversification across several posterior cortical neurodegenerative syndromes, many of which share Alzheimer’s disease pathology.^23,92,153,154^ Shifting the focus to reveal the importance of parietal integrity in Alzheimer’s disease will move the field a step closer to achieving a full understanding of the interplay between neurodegeneration and cognitive dysfunction in multiple clinical variants of Alzheimer’s disease.

As we make progress on the aforementioned steps, it seems likely that we will uncover deeper layers to the lvPPA cognitive and behavioural profile. Therefore, a final, important step forward is to build integrative frameworks of cognitive and brain mechanisms that underpin the broader symptom complex of lvPPA, with relevance to other neurodegenerative dementia syndromes affecting the posterior neocortex. This larger programme requires a two-pronged approach, with each step informing the other. What we first need is a better understanding of how psychological and pathophysiological changes in lvPPA relate to changes in local neural populations and their neurochemistry, genetics and neuropathology, and neuroimaging in the syndrome. This will allow integration of new knowledge regarding brain, behaviour and biological changes in lvPPA with current neurocognitive models. Next, testing the stability and interpretability of these renewed models requires international, multi-centre studies to establish large lvPPA cohorts with deep neuropsychological and neuroimaging phenotyping, and identify candidate neurophysiological biomarkers in the diagnostic process. We acknowledge that this future direction is ambitious and requires navigating recruitment challenges and nosological disagreements. However, we hope that our framework can aid this process by accommodating terminological discrepancies and diverse clinical-cognitive presentations within a multidimensional space, and potentially offer a common research framework to springboard future efforts towards a complete understanding of the lvPPA phenotype.

## Abbreviations

IPL =: inferior parietal lobe
lvPPA =: logopenic variant primary progressive aphasia
nfvPPA =: non-fluent/agrammatic variant PPA
PPA =: primary progressive aphasia
svPPA =: semantic variant PPA
TPJ =: temporoparietal junction
